# A Systematic Review of Self-Report Measures of Negative Self-Referential Emotions Developed for Non-Clinical Child and Adolescent Samples

**DOI:** 10.1007/s10567-020-00339-9

**Published:** 2021-02-05

**Authors:** Hajra Ashra, Christopher Barnes, Edward Stupple, Frances A. Maratos

**Affiliations:** School of Psychology, College of Health, Psychology & Social Care, University of Derby, Derby, UK

**Keywords:** PRISMA, Mental health, Youth, Wellbeing, Child practitioners

## Abstract

The crisis in child and adolescent mental health and wellbeing has prompted the development of school and community-based interventions to tackle negative emotions towards the self. Providing an evidence-base for such interventions is therefore a priority for policy makers and practitioners. This paper presents the first systematic review of self-referential and self-report measures of negative emotions for use with non-clinical child/adolescent populations, and evaluation of their psychometric properties. A systematic search of electronic databases and grey literature was conducted. Peer reviewed articles that introduced a new measure or included psychometric evaluation of a negative self-referential emotion for children and/or adolescents were identified. Study characteristics were extracted, and psychometric properties rated using internationally recognised quality criteria. Initially, 98 measures designed for evaluating children and adolescents’ negative self-referential emotions were found. Measures were primarily excluded if they were intended for clinical diagnosis or did not focus on self-referential emotions. The remaining eight measures (Brief Shame and Guilt Questionnaire; Self-Consciousness Scale-Children; Shame and Guilt Scale for Adolescents; Test of Self-Conscious Affect- Adolescents; The Child-Adolescent Perfectionism Scale [CAPS]; Child and Adolescent Dysfunctional Attitudes Scale Revised; Children Automatic Thoughts Scale [CATS]; Negative Affect Self-Statement Questionnaire) were organised into domains consisting of self-conscious emotions, self-oriented perfectionism and negative self-cognitions. Psychometric quality ratings identified the CAPS (Flett et al. in J Psychoeduc Assess 34:634–652, 2016) and the CATS (Schniering and Rapee in Behav Res Ther 40:1091–1109, 2002) as having the strongest psychometric qualities. However, all reviewed measures lacked full evaluation of essential psychometric properties. Our review revealed a paucity of self-referential emotional measures suitable for assessing adverse negative self-referential emotions in general child and adolescent populations. Measures suitable for use in non-clinical samples were identified, but these require further evaluation and/or new scale developments are needed. The psychometric findings and methodological issues identified will guide researchers and practitioners to make evidence-based decisions in order to select optimal measures.

**Supplementary Information:**

The online version of this article (10.1007/s10567-020-00339-9) contains supplementary material, which is available to authorized users.

## Introduction

Children and young people’s mental health is in a state of crisis across the world (e.g., Bruha et al. 2018; Gunnell et al. 2018). It has been identified as a priority by many governments (e.g., Department of Health England, UK 2017; Ministry of Health, New Zealand 2019) and exacerbated by COVID-19 (Torres-Pagán and Terepka 2020; Power et al. 2020). Negative emotions and negative cognitions about oneself play a critical role in the onset and duration of emotional problems (Gilbert 2000). This includes negative emotions related to personal failure, self-blaming emotions, self-conscious emotions (shame and guilt), self-criticism, self-thinking, and judgments (Gilbert and Irons 2005). These negatively biased ‘self-referential’ emotions/cognitions are of importance, as they appear to be a significant risk factor for the development of depressive disorders (LeMoult et al. 2017). In addition, the negative self-referential emotions of shame and self-criticism are associated with a range of psychological difficulties, including various forms of depression, substance misuse, eating disorder, social anxiety, and psychosis (see for example Gilbert and Irons 2005; Zuroff et al. 2005; McIntyre et al. 2018 for reviews). Most recently, Liu et al. (2020) have demonstrated that preadolescent children at high risk for depression demonstrate differential prefrontal brain activity when engaged in self-referential thinking as compared to their low-risk counterparts. They suggest that such activity might ‘*portend maladaptive emotion regulation that eventuates in depression*’ (p. 434). For these reasons, implementing effective interventions that prevent, reduce and enable individuals to regulate self-referential negative emotions is essential for tackling the global mental health crisis. However, to enable this, the use of robust, appropriate instruments to measure negative self-referential emotions is a necessity.

## The Self and Mental Health in Youth

Self-referential emotions are particularly important during childhood and adolescence, as it is during these periods that individuals begin to develop the capacity to refer to self-emotions and cognitions (Harter 1986); as opposed to more concrete emotions (e.g., linking particular emotions to particular situations). The transition between childhood and adolescence is the time when questions around self-identity are formed; for example, “Who am I?’ or “Am I good enough?” (Harter 1982). Whilst it is possible to find various definitions of self-referential emotions (Becker et al. 2011; Zinck 2008), of key importance, self-referential emotions as compared to the generic (or outward) measurement of emotion, include reference to the self. For example, in the case of anger “I get angry with myself” or “I have become so angry with myself that I want to hurt myself” are self-referential emotions, whereas “I am angry” or “I am often angry with others” are not. In the latter example the subject is self-referential, but the reference an external object. The demonstration of basic emotions, such as anger (or ‘I am angry’), are evident from birth. Self-referential emotions, in contrast, begin to develop with the advent of theory of mind. That is, from around ages 4 to 5 in typically developing children (Zinck 2008). While self-evaluations are important elements of general wellbeing, when evaluations represent frequent negative emotions about the self—e.g., “I hate myself” or “I feel like I am a failure”—(i.e., both the subject and the reference are self-directed), adverse consequences may be observed. For example, in addition to depression and eating disorders (see also Beck 1987; Vitousek 1996, respectively), frequent negative self-referential emotions have been linked to suicide-related responses (Nock and Kazdin 2002), generalised anxiety disorder (Wells 2004) and low self-esteem (Verplanken et al. 2007). In childhood/adolescence, moreover, the development of the negative emotions of shame and self-criticism have been linked to vulnerability to various psychopathologies/mental disorders (Gilbert and Irons 2005; Xavier et al. 2016).

In the UK alone, one in ten primary aged children, and one in seven secondary aged pupils are reported to suffer from a mental disorder, and this number is increasing (NHS 2018). Similar statistics are reported in the US with 7.1% of children aged 3–17 years (approximately 4.4 million) diagnosed with anxiety, and 3.2% of children aged 3–17 years (approximately 1.9 million) diagnosed with depression (Centre for Disease Control 2020). Worldwide, the World Health Organisation (2020) reports that 10–20% of children and adolescents experience mental disorders which, if left untreated, can severely influence development attainments, educational attainments and quality of life. Patel et al. (2007) further state that poor mental health not only contributes to lower educational achievement, but also increased rates of engagement in health risk behaviours (such as substance abuse and poor sexual health), self-harm and suicide. In their review, they note interventions that promote mental health prior to disorder onset are necessary. They suggest that whilst drug and psychotherapeutic interventions prove efficacious for mental disorder treatment, demand for such services exceeds availability. Consequently, they argue that practitioners and policy makers need to focus intervention dissemination through community-based channels, such as educational settings. This is a proactive approach to wellbeing aimed at reducing the burden of mental health by engaging in preventative/pre-emptive mental health promoting strategies. As such, there is a need to be able to identify groups of children and young people, without a specific mental health diagnosis or statement, who are experiencing reduced levels of wellbeing.

## The Measurement of Wellbeing in Youth

Despite interest in child and adolescent emotional wellbeing, and the ramifications of negative self-emotions, inconsistencies in defining child and adolescent wellbeing, including the variety of indicators and measures available, have created a confusing and contradictory research base (Pollard and Lee 2003). As the construct of emotional wellbeing is multi-faceted, a variety of options to measure wellbeing across positive and negative emotional indicators are available for child and adolescent populations. This array of measures indicates not only advancement in the field, but also a lack of consensus regarding optimal indicators of wellbeing. For instance, Žukauskienė et al. (2015) identified *104* measures designed to evaluate a range of emotions in child/adolescent samples that could be used with children and adolescent populations. However, the negative emotional wellbeing measures they identified were either disorder specific measures, focused on psychological problems, such as depression (for example, the Children’s Depression Inventory (CDI; Kovacs 1981) or multi-dimensional measures covering a variety of wellbeing indicators (e.g., quality of life, life satisfaction, self-perception and self-concept, social emotion, coping, expression of emotions and emotion regulation). Although these measures provide meaningful insights into child/adolescent emotional wellbeing, focus on *self-referential* emotions i.e., emotions directed toward the child/adolescent themselves was not a criterion of their review.

Most recent reviews and databases of measures of emotion for children and adolescents have further focused on their use in clinical practice (Bentley et al. 2019; Deighton et al. 2014; Han 2009; Simmons et al. 2015). For example, Simmons et al. (2015) provided a review of measures suitable for depression, and Han (2009) a review of those suitable for anxiety. Of those reviews identifying measures suitable for use in non-clinical/community-based child and adolescent populations, again, the focus has often been on evaluating a child or adolescent’s ability to recognise emotions of others (e.g., Social and Emotional skills) or psychosocial emotions (Tsang et al. 2012), rather than self-referential emotions. Additionally, previous systematic reviews and guidance (e.g., Public Health England 2015), as well as open-access databases (e.g., CORC 2016; SPECTRUM Database 2011), often exclude specific measures of negative emotions (e.g., measures of anger, sadness, perfectionism, shame and guilt) and provide little indication on which measure is most appropriate to target certain emotions, particularly with respect to their psychometric qualities and properties (De Boer et al. 2004).

Thus, whilst a plethora of validated wellbeing measures for use in youth populations exist, those suitable for use in community-based samples is often lacking. In adopting a pro-active approach to mental-health and wellbeing, a review of measures is needed that enables exploration of vulnerability to mental-health disorders rather than the identification of such disorders. Specifically, negative self-referential emotional measures, given they are an identified risk factor portending a variety of psychopathologies.

## Study Aims, Objectives and Rationale

Since schools and community initiatives provide a critical context in which young people develop their sense of self, interventions are increasingly used within these settings to investigate and regulate negative self-referential emotions (Fazel et al. 2014). Moreover, educational polices, such as the UK “2020 Vision” require evidence-based practice of mental health interventions in school settings (Department for Education 2018; see also NICE quality standard 2018). With the rapid growth of emotional-wellbeing measurements designed for use with children and adolescents (Pollard and Lee 2003), finding suitable self-referential emotional measures, so that regulation of negative affect can be investigated, is a significant challenge. Indeed, given the paucity of either an overview or review of negative self-referential emotion measures, but their clear importance for assessing mental health and wellbeing, the purpose of this review is to: (i) identify current negative self-referential emotional measures fit for use in school and community-based interventions (i.e., those not used for the primary purpose of diagnosing a disorder), (ii) report evidence of their psychometric properties, to (iii) enable educators, researchers and practitioners to locate the most reliable and valid measures.

As Orth and Wyk (2020) note, measures aimed at diagnosing (or screening for) mental health problems are useful in contexts where child and adult mental health services are well developed and supported. However, in low to middle income countries, lack of policy development, resources and services result in many challenges associated with the use of such scales (e.g., the sustainability of adolescent mental healthcare services per se). Additionally, as such interventions are often delivered by staff who rarely have specialised clinical training to diagnose children and young people (Severson and Walker 2002), diagnostic and clinical measures may not be suitable. Finally, a focus on clinical/diagnostic measures can result in problems of floor effects (i.e., limited score ranges), especially when using clinical measures with non-clinical populations. For these reasons, and in accord with a proactive approach to mental health (Patel et al. 2007; Orth and Wyk 2020), self-report measures specific to a diagnosis and/or related to DSM diagnostic classification were excluded from the present review.

The focus of the review further included only those measures that allow for self-report, rather than observation or experimental paradigms etc. This was because: (i) self-referential emotions are internalised emotions that are not always easily detectable by third party observations (Reynolds 1992); and (ii) self-report measures are usually fairly easy to administer, even by non-experts. Framework proposed by Terwee et al. (2007) and Mokkink et al. (2010), were used to appraise the psychometric properties of included measures. Terwee et al. and Mokkink et al. have developed comprehensive quality checklists that can be used when designing, selecting or evaluating the properties of outcome measurement instruments, in order to enhance outcome measurement development and selection. Finally, and consistent with our definitions of self-referential emotions presented previously, we defined negative self-referential emotions as those referenced in the context of the *self only*. That is, they are negative emotions composed of a subject and reference towards the self, e.g., ‘I often get angry with myself’, as opposed to ‘I often get angry with others’ where the reference is non-self.

## Methods

The PRISMA statement has been used to guide the methodology and reporting of this systematic literature review. The PRISMA statement (2009) contains a 27-item checklist of elements considered to be essential for ensuring transparency in undertaking and reporting systematic literature reviews.

### Stage 1: Identification of Measures

A list of self-referential negative emotions was derived based on: The Positive and Negative Affect Scale-Extended Form measure of emotions (PANAS-A, Watson and Clark 1994); emotional wellbeing literature (e.g., Pollard and Lee 2003) and collective knowledge of the research team. To ensure the list was exhaustive, experts in the field were also contacted allowing additional items to be included in the search. This stage generated a list of final search terms which were used to run searches across each of the databases (Table 1).

**Table 1:** Categories and related terms for the systematic review

Category	Related search terms
1. Negative self-referential emotions	Shame OR Guilt OR Embarrassment OR Jealousy OR Envy OR Self-criticism OR Self-hating OR Self-loathing OR Perfectionism OR Pessimism OR Disgust OR Loneliness OR Pity OR Anger OR Sad* OR Fear
2. Population of interest	Child* OR Young people OR Adolescence*or Pupil* OR Preadolescence*
3. Measurement	Measure* OR Scale* OR Inventory* OR Assessment* OR Questionnaire* OR Instrument* OR Inventory* OR Survey* OR Test* OR Evaluation* OR Screening OR Psychometric*
4. Psychometric properties	Development OR Reliability* OR Validity

A systematic search was conducting using the Library Plus electronic database, which includes a range of databases in areas of behavioral science and mental health (e.g., Embase, ERIC, PsycINFO and PubMed). The search was then expanded to secondary searches in grey literature through Google Scholar. A web search was also conducted using ‘Google’ to identify additional measures not identified in the electronic database searches. Database searches were conducted between the period of June 2018 to February 2020. The initial search strategy was focused upon including all journal articles published in the English Language. Searches were undertaken by three members of the research team (HA, CB & FM) and filtered by the first author (HA). The search strategy was split into four search terms and their related items. Search terms were limited to abstract and peer-reviewed journal articles only. Additionally, to ensure the review was thorough, websites that review emotional measures for children and adolescents (CORC and SPECTRUM) were also reviewed by HA. Scale/paper authors were also contacted when, for example, full item lists or psychometric properties were not stated in the paper.

### Stage 2: Eligibility Criteria

#### Title and Abstract Screening

Basic filtering was applied through reading the title and, where the title was not sufficient, the abstract, to identify peer reviewed studies citing the psychometric properties of the measures designed. Papers were excluded if any of the following criteria were applicable: (a) not a scale development paper nor a validation paper; (b) the scale did not measure a negative ‘self-referential’ emotion; (c) the scale was not primarily validated for use with non-clinical samples; and/or (d) the article/scale was not written in English. To capture the most comprehensive review of negative self-referential emotional measures within the field, non-English measures translated/validated for an English-speaking population were also eligible for this review. To ensure inter-rater reliability at this stage, every rater evaluated a random selection of 10% of the resultant search studies (i.e., HA, CB, ES & FM) and any disagreements were resolved through discussions. The observed Kappa was 0.76 for the title and screen review. As a rule of thumb, values of Kappa from 0.40 to 0.59 are considered as moderate, 0.60 to 0.79 substantial and 0.80 outstanding (Landis and Koch 1977).

#### Full Text Extraction Screening

This involved reading the relevant abstract and/or article and applying the below inclusion/exclusion criteria to guide full-text extraction screening.

### Inclusion Exclusion Criteria

1. The article must be a peer-reviewed publication (journal article) only.
2. The focus of the article must be on a self-report measure. Second person measures (e.g., teacher report of a pupil’s emotional state) were excluded.
3. The article describes initial development or validation of the self-report measure/s and must report psychometric data. Behavioural, observational and physiological measures were excluded.
4. The article must describe a self-referential emotion measure, but this can be within a specific negative measures of emotion. Non-self-referential measures (e.g., emotion regulation) and generic mental health measures were excluded.
5. The study sample includes children in the age range of 8–18.
6. The sample is non-clinical OR indicates a “General Population” (e.g., community-based).
7. The scale must include at least one negative self-referential emotion subscale and/or at least one negative self-referential emotion item (i.e., an item/scenario where the *subject* and the *reference* are directed toward the *self)* in the final measure.
8. The primary use of the measure is not as a diagnostic tool (e.g., clinical DSM criteria).
9. All items in the measure are provided in English.

### Stage 3: Extracting Data

The details of the included measures were transferred and organised into an Excel spreadsheet where descriptive data extracted from the publications including characteristics of included studies (e.g., country), characteristics of the sample (e.g., population age), general characteristics of the measures (e.g., negative self-referential domain into which the items could be classified) and psychometric properties assessed (e.g., internal reliability), were noted. To ensure consensus of the data extraction process, a standardisation procedure was performed in which the four raters (i.e., HA, CB, FM & ES) performed extraction on four full papers together. The lead reviewer (HA) then individually extracted the remaining measures. Once completed, retained measures were re-screened by a second rater individually (CB, FM or ES) to ensure consistency. 100 percent inter-rater agreeability was observed.

### Stage 4: Quality of Psychometric Properties Assessment (Terwee et al. 2007)

The quality criteria used in this review were based on Terwee et al. (2007), Mokkink et al. (2010) and De Leeuw (2011). These were the nine criteria proposed by Terwee et al. (2007): content validity, internal consistency, criterion validity, construct validity, reproducibility agreement, reproducibility reliability, responsiveness, floor and ceiling effects and interpretability (for review, see their Table 1, p. 39). An additional criterion from the COnsensus-based Standards for the selection of health Measurement INstruments (COSMIN, Mokkink et al. 2010) was included to examine cross-cultural validity. Finally, we also included a readability criterion, because assessing readability is an easy and efficient indication of measure ‘ease of use’ for a targeted age group (De Leeuw 2011). This resulted in a total of eleven measurement properties, which are described in full in the relevant results section.

In the current systematic review, each psychometric property was initially rated according to the system of Terwee et al. (2007) and rated as positive (+) if the results meet their specified criteria, negative (−) if they fell short of their standards, intermediate (?) if the results were not consistent with criteria and (0) if no information was found for that psychometric property. However, in accord with Wittkowski et al. (2017), to obtain numeric values, the above ratings were coded so that a “ + ” = 3, “-” = 2, “?” = 1 and “0” = 0. Thus, each included measure could achieve a total score ranging from 0 to 27, with higher scores indicating better psychometric qualities. Note that this score should only be used as a guide. This is because it can incorrectly imply that all measurement properties are equally important; yet readers may wish to consider choice of measure based on the presence of a specific criteria.

## Results

### Included/Excluded Studies

**Fig. 1:**
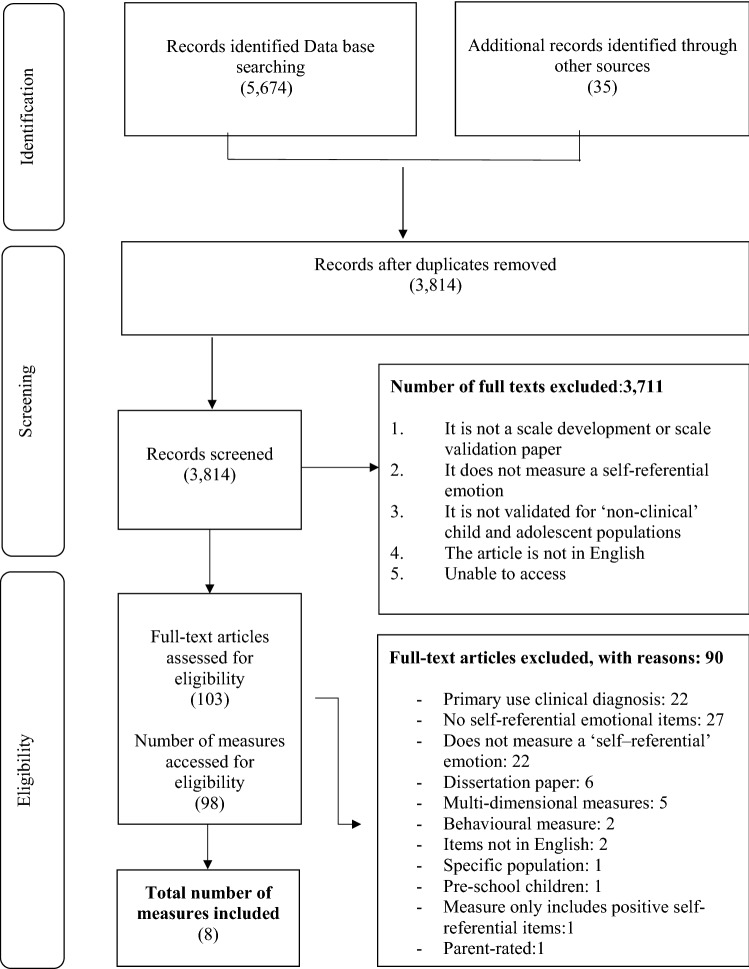
Flow chart according to PRISMA showing the search and selection process of studies related to negative self-referential emotional measures for children and adolescents

A flow chart of the search and selection process is presented in Fig. 1. Our search identified a total of 5674 records and the secondary grey literature search generated an additional 35 records that met initial inclusion criteria, resulting in 5709 hits. A total of 3814 remained following removal of duplicates. Following title and abstract screening, 103 full text articles reporting on 98 different measures were assessed for eligibility. Of these, 90 measures were excluded for the following reasons: 22 measures were primarily used for clinical diagnosis (e.g., Child Depression Inventory; Kovacs 1992); 27 measures did not have any self-referential emotional items (e.g., Holistic Anger Rating Scale; Mani et al. 2018); 22 measures did not measure a ‘self-referential’ emotional domain (e.g., Social Anxiety Questionnaire for Children; SAQ-C; Caballo et al. 2016); six measures were dissertation papers (e.g., Interpersonal Guilt Questionnaire; Mulherin 1998); five measures were multidimensional measures (e.g., Strengths and Difficulties Questionnaire, Goodman 2001); two measures did not include items in the English language (e.g., The Aggression Questionnaire for Spanish preadolescents and adolescents; Santisteban and Alvarado 2009); two measures were behavioural measures (e.g., Anger Behavioural observation; Rohlf and Krahé 2015), one measure was parent rated (Parent rated Life Orientation Test for children; Lemola et al. 2010); one measure was in a specific population (Guilt and Shame Questionnaire—for Adolescents of Parents with a Mental Illness; Bosch et al. 2020); one measure was for pre-school children (Children’s Moods, Fears and Worries Questionnaire; Bayer et al. 2006) and, finally, one measure included a self-referential subscale, but the sub-scale items captured positive self-emotions (KINDL, Ravens-Sieberer and Bullinger 1998). This led to the final inclusion of eight negative self-referential emotional measures. For a full list of excluded measures and reasons for exclusion please refer to Supplementary Table 1.

#### Constructs Captured in Included Measures of Negative Self-referential Emotions

Details of final scales included in the review can be found in Table 2. Constructs captured by the included scales illustrate a diversity of conceptualisation of negative self-referential emotions, which were grouped into three over-arching themes/domains:

**Table 2:** Study and measurement characteristic of the assessment of children’s and adolescent’s self-referential negative emotions

Measurement Author (date) and country	Self- referential emotion assessed	Purpose of the study	Questionnaire purpose, item overview, total items [T] and percentage [%] self-referential	Study population [N] Age (range [R] and/or mean [M] standard deviation [SD]	Response option	Example item
Brief Shame and Guilt Questionnaire (BSGQ) Novin and Rieffe (2015) Netherlands, Denmark	Self- conscious emotions	To present and validate a brief questionnaire to measure shame and guilt-proneness in children	A scenario-based measure of shame and guilt proneness. The items involve various situations intended to elicit shame and guilt. It is comprised of two sub-scales: Shame = 6 items; Guilt = 6 items. T = 12 items. Scenario items 100% Self-referential	Total sample: *N* = 219; *R* = 8.04–14.24 years; *M* = 10.17; SD 1.12	3-Point scale rated as: 1 = not at all; 2 = A little; 3 = A lot	“You are standing in front of the class. You have to give a talk. Everyone is looking at you. You forget what you wanted to say” (P. 57)
Self-Consciousness scale-children (SCS-C) Two versions: Children version (JUNIOR82). Adolescents version (TEENTEST81) Abrams (1988) UK	Self- conscious emotions	To modify and validate the original SCS (Fenigstein et al. 1975) for children and adolescents, as well as report factor structure reliability and validity	A questionnaire to measure (negative) self-consciousness. The items capture self-criticism, recognition of one’s positive and negative attributes; introspective behaviour; a tendency to picture, or imagine, oneself; awareness of one’s physical appearance and presentation; and concern over appraisal by others. It is comprised of two versions. The Children’s ‘JUNIOR82′: Two sub-scales: Private = 5; Public/Conformity = 3. T = 8 items. 12% Self-referential. Adolescent ‘TEENTEST81′: Four sub-scales: Private = 5 items; Public = 4 items; Social Anxiety = 1 items; Personal attitudes to group cohesiveness = 5 items. T = 15 items. 43% Self-referential	Total Sample *N* = 246 Children’s JUNIOR82: *N* = 63; *R* = 10–11 *M* = NS; SD NS Adolescent TEENTEST81: *N* = 183; *R* = 12–13; *M* = NS; SD NS	Strength of agreement for each item rated from 1 ‘strongly agree’ to 7 ‘strongly disagree’	“I never scrutinize myself” (P.12)
Shame Scale for Adolescents (EVA) and Guilt Scale for Adolescents (ESCA) Laskoski et al. (2013) Brazil	Self- conscious emotions	To develop and validate measures to assess shame and guilt. To test the empirical independence of the constructs	A questionnaire measure to access shame and guilt as two theoretically different moral emotions. The items include statements representing self-feelings or behaviours. Shame items focus on fear of judgment, inferiority and negative evaluation from others**.** Guilt items focus of feelings of regret and remorse for actions. It is comprised of two scales, the EVA = 8 items; the ESCA = 11 items. T = 19 items. 56% self-referential	Total sample: *N* = 580; *R* = 13–18 years; *M* = 16.0; SD 1.19	5-Point scale rated from 1 = never occurs to 5 = always occurs	“I feel bad when I want to do something that I know is not right” (P. 174)
Test of self- conscious affect- adolescents (TOSCA-A) Watson and Gullone (2016) Melbourne, Australia	Self- conscious emotions	To examine the application and psychometric properties of the TOSCA-A for child and adolescent populations	A scenario-based measure to capture guilt proneness and shame proneness. It comprises of 10 negative scenarios and 5 positive scenarios, that would be likely events experienced by young people. T = 15 items. Scenario items 100% Self-referential	Total sample: *N* = 1261; Child sample: *N* = 699; *R* = 8.12–11.99; *M* = NS; SD = NS. Adolescents Sample: *N* = 562; *R* = 12.01–16.15; *M* = NS; SD = NS.	5-Point scale with ratings of: 1 = very unlike me; 2 = a little unlike me; 3 = maybe (half and half); 4 = a little like me; 5 = very like me)	At lunchtime you trip and spill your friends drink” Shame response: “I would be thinking that everyone is watching me and laughing” Guilt response: “I would feel very sorry, I should have watched where I was going” (P. 3)
The Child-Adolescent Perfectionism Scale (CAPS) Flett et al (2016) Toronto, America	Perfectionism	To describe the psychometric properties of the CAPS. To provide descriptive results for the CAPS from several samples, including clinical children and adolescents and children/adolescents in China, Israel and Russia	A measure of perfectionism. It includes a self-oriented perfectionism subscale containing motivational and cognitive self-referent content focused on perfectionistic standards and self-evaluations in ability (or inability) to attain these standards. It consists of two sub-scales. Self-Oriented Perfectionism = 12 items; Socially prescribed Perfectionism = 10 items. T = 22 items; 32% self-referential	Total sample: *N* = 2943 Study 1: Non-clinical child/adolescent’s *N* = 247; *R* = 8–18 years, *M* = 13.28; SD 3.23. Study 2: Non-clinical child/adolescent’s: *N* = 796; *R* = Elementary or High school children; *M* = 13.45; SD 4.05 Study 3: Non-clinical adolescent’s: *N* = 553; *R* = NS; *M* = 13.02; SD 0.38 Additional samples: clinical sample: *N* = 279; *R* = 8–20; *M* = 13.5; SD NS 3 Non-clinical samples China: (1) Grade 5: *N* = 218; *R* = NS; *M* = 12.19; SD NS. (2) Grade 8: *N* = 172; *R* = NS; *M* = 14.17; SD NS. (3) High School: *N* = 242; *R* = NS; *M* = 17.98; SD NS 1 Non-clinical adolescents Israel:*N* = 283; *R* = NS; *M* = 16.40; SD 1.57 Non-clinical adolescents Russia: *N* = 153: *R* = NS; *M* = 15.00; SD 1.05	5-Point scale with ratings of: 1 = False—not at all like me; 2 = Mostly False; 3 = Neither true or false; 4 = Mostly true; 5 = very true of me	“I get mad at myself when I make a mistake” (P. 16)
Child and Adolescent Dysfunctional attitudes scale (CDAS-R) McWhinnie et al. (2009) Canada	Perfectionism	To modify and validate the original 40-item DAS (Weissman & Beck, 1978) for use with child/adolescents. To report factor structure/psychometric properties of the CDAS-R	A questionnaire measure aimed at accessing maladaptive thinking, through negative views of self and perfectionism. It consists of two sub scales. Self-critical perfectionism = 9 items; Personal standards perfectionism = 6 items. T = 15 items. 20% self-referential	Total sample: *N* = 454 Children sample: *N* = 130:*R* = 8–9; *M* = 8.8; SD 0.38 Adolescents sample: *N* = 184; *R* = 12–13; *M* = 12.8 SD 0.62. Clinical sample: *N* = 140; *R* = 6–14; *M* = NS; SD NS	4-Point scale with ratings of: 0 = Never true; 1 = Most of the time true; 2 = Just sometimes true; 3 = Always true	“If I make a mistake, I should get mad at myself” (P. 296)
Children’s automatic Thoughts Scale (CATS) Schniering and Rapee (2002) Australia	Negative Self-cognitions	To describe the development, factor structure and psychometric properties of the CATS in child and adolescent populations	The CATS is designed to assess a wide range of negative self-thoughts across both internalizing and externalizing problems. It comprises of four sub-scales. Physical threat = 10 items; Social threat = 10 items; Personal failure = 10 items; Hostility = 10 items. T = 40 items. 20% self-referential	Total sample: 893; Sample 1: Non-clinical: *N* = 762; *R* = 7–16; *M* = 13.38; D = 2.00. Sample 2: Clinical: *N* = 131; *R* = 7–1; *M* = 12.01; SD 2.84	5-Point scale with items ranging from: 0 = not at all to 4 = all the time	I hate myself” (P. 1096)
Negative Affect Self-statement Questionnaire (NASSQ) Ronan et al. (1994) United States	Negative Self-cognitions	To report the development of two separate measures: one for children aged 7–10 and one for adolescents 11–15 years. To report validity and reliability	The NASSQ assesses referential cognitive experiences of negative affect based on thoughts that “pop” into child and adolescent minds. Items represent a wide range of negative self-referential statements. It is comprised of two versions: The NASSQ ages 7 to 10: Two subscales: Anxiety-specific (AS) = 11 items; Depression-specific (DS) = 3 items. T = 14 items. 21% Self-referential. The NASSQ ages 11 to 15: Three subscales: Anxiety-specific (AS) = 21 items; Depression-specific (DS) = 8 items; Negative Affect (NA) = 10 items. T = 39 items. 15% self-referential	Total sample: *N* = 569 Sample 1: Children Sample: *N* = 241; *R* = 7–10; *M* = 9.00; SD 0.88.Adolescent Sample: *N* = 301; *R* = 11–15; *M* = 11.95; SD 0.94. Sample 2: Clinical *N* = 27; *R* = 8–15; *M* = NS; SD NS	5-Point scale rated as:1 = Not at all; 2 = Sometimes; 3 = fairly often; 4 = often; 5 = all the time	NASSQ for 7-to-10 year old’s “I thought I would fail” ( P. 516) NASSQ for 11-to-15 year old’s “I was afraid I would make a fool of myself” (P. 518)

*NS* not stated

Domain 1: Captured scales measuring negative ‘self-conscious’ emotions. This included emotions of shame, guilt and the self-conscious emotions more broadly (e.g., self-criticism and recognition of one’s negative attributes). In these scales, shame is defined as self-referential feelings of worthlessness, inability, powerlessness and incompetence (the Shame Scale for Adolescents; EVA); negative evaluation of the self (the Brief Shame and Guilt Questionnaire; BSGQ); or negative evaluation of the self with a focus on unworthiness (Test of Self-Conscious Affect- Adolescents; TOSCA-A). Guilt is defined as feelings of remorse and regret (Guilt Scale for Adolescents; ESCA); or ‘ones negative evaluations of one’s behaviour and transgression’ (the BSGQ) following a specific action or behaviour (the TOSCA-A). The self-conscious scale-children (SCS-C) represented a more diverse suite of negative shame/guilt emotions, but based on private and public self-consciousness, where private is defined as attending to one’s inner thoughts and feelings, and public as a general awareness of the self as a social object that has an effect on others.

Domain 2: Negative cognitions. This included negative emotions and thoughts related to failure, self-dislike/hate, worthlessness; but broader in scope than the constructs of shame and guilt. For example, the Children’s Automatic Thoughts Scale (CATS) includes a failure sub-scale with items pertaining to worthlessness, self-blame/failure, pessimism, hopelessness and self-hate. Similarly, the Negative Affect Self-Statement Questionnaire (NASSQ) includes items representing feelings of failures, shame, pessimism, self-criticism and self-devaluation.

Domain 3: Self-oriented perfectionism. This included negative self-referential emotions related to perfectionism, including having exceptionally high personal standards and being driven to achieve these standards (the Child-Adolescent Perfectionism Scale; CAPS; and the Child and Adolescent Dysfunctional Attitudes scale Revised; CDAS-R), including mistake making. However, the CDAS-R also included items mapping onto the construct of self-criticism.

In sum, whilst we have grouped measures into three domains based on author positioning of the measures, suggested content and our own review of items, it is important to note that items within a specific measure often elicited a wide range of negative self-referential emotions. These on occasion crossed domains. For example, the CDAS-R perfectionism measure (domain 3) included items that mapped onto the ‘negative cognitions’ measures (domain 2), and measures of negative cognitions included items that mapped onto shame and guilt, captured in the ‘self-conscious emotions’ measures (domain 1).

#### Study Demographics, Number of Items, Sub-scales and Response Option Summaries

Study sample size ranged from *N* = 219 (BSGQ) to *N* = 2943 (CAPS), with most measures for use with both children and adolescents (*n* = 5; BSGQ, CAPS, CATS, CDAS-R & TOSCA-A). Within non-clinical samples, age ranged from 7 to 18 years. Two measures (NASSQ & SCS-C) included separate versions of the measure, one for children and one for adolescents. One measure (EVA/ESCA) was specifically for adolescents. The CAPS covered the widest age range in a non-clinal sample (8–18 years). The greatest number of self-report measures came from the United States of America/Canada (*n* = 3), with the remaining measures developed and/or validated in the United Kingdom (*n* = 1), Australia (*n* = 2), the Netherlands (*n* = 1) and Brazil (*n* = 1). One measure (CAPS) included samples from several countries, including China, Israel and Russia. The EVA/ESCA was translated from a different language into English.

Number of items ranged from 8 to 40 (*M* = 19.9, SD 10.4). The SCS-C had the fewest items (8 for the child version; 10 for the adolescent version), while the CATS had the highest number of items (40 items). All measures included multiple subscales, ranging from two (BSGQ, EVA/ESCA, NASSQ [child version], TOSCA-A, CDAS-R, SCS-C Junior82 & CAPS) to four (SCS-C & CATS). All eight measures used a Likert type scale ranging from 3- (BSGQ) to 7-points. Most measures adopted a 5-point scale (CATS, NASSQ, TOSCA-A, EVA/ESCA & CAPS). The SCS-C used a visual analogue scale, where participants are asked to note strength of agreement for each item by placing a cross on a line labelled from ‘strongly agree’ to ‘strongly disagree’. This is later scored as a 7-point scale. Two measures adopted a scenario-based method presenting participants with scenarios to elicit self-referential emotions (BSGQ & TOSCA-A).

### Overview of the Psychometric Characteristics of Measures

The results of the quality assessments across all quality indicators (i.e., individual scores for each criterion as well as an overall quality score) are presented in Table 3. The overall score consists of all properties apart from cross-cultural validity because this criterion was only applicable to two measures. These were the CAPS and EVA/ESCA. In summary, all studies reported internal validity (*n* = 8; 100%) and the majority reported content validity (*n* = 6; 75%) and criterion validity (*n* = 5; 62.5%). Fewer studies reported agreement (*n* = 4; 50%), construct validity (*n* = 3; 37.5%), and/or interpretability (*n* = 3; 27.2%). Finally, no measure provided information on responsiveness nor floor/ceiling effects. Consequently, all measures scored 0 for these last two properties.

**Table 3:** A summary of psychometric quality assessment achieved by each of the eight negative self-referential measures of emotion

Measure	Content validity	Internal consistency	Criterion validity	Construct validity	Agreement	Responsiveness	Floor and ceiling	Interpretability	Readability	Cross cultural validity*	Overall Score (0–27)
1. CAPS	1	3	2	1	1	0	0	1	3	2	12
2. CATS	3	3	0	0	3	0	0	1	0	–	10
3 TOSCA-A	1	3	2	3	0	0	0	0	0	–	9
4. NASSQ	2	1	3	0	1	0	0	1	0	–	8
5. BSGQ	0	3	2	3	0	0	0	0	0	–	8
6. CDAS-R	0	3	2	0	0	0	0	0	0	–	5
7. EVA & ESCA	2	3	0	0	0	0	0	0	0	0	5
8. SCS-C	1	2	0	0	2	0	0	0	0	–	5

*CAPS* Child-Adolescent Perfectionism Scale (Flett et al. 2016), *CATS* Children automatic Thoughts Scale (Schniering and Rapee 2002), *TOSCA-A* TOSCA-A (Watson and Gullone (2016), NASSQ Negative Affect Self-statement Questionnaire (Ronan et al. 1994), *BSGQ* Brief Shame and Guilt Questionnaire for Children (Novin and Rieffe 2015), *CDAS-R* Child and Adolescent Dysfunctional Attitudes Scale Revised (McWhinnie et al. 2009), *EVA/ESCA* Shame and Guilt Scale for Adolescents (Laskoski et al. 2013), *SCS-C* Self-Consciousness scale-children (Abrams 1988)

– not applicable

*Not included in overall score

### Content Validity

Only one measure, the CATS, received the highest rating of three for content validity, indicating the measurement aim, target population, concepts being measured, and process of item selection were clearly described by the authors. Two measures (EVA/ESCA & NASSQ) scored two, indicating target population involvement in item selection, but no expert opinion. A total of three measures (TOSCA, SCS-C, CAPS) scored one. This indicated that a clear description of the aforementioned aspects was lacking, that the target population was not involved, and/or that the design and methods used to ensure content validity was doubtful or lacking item selection information. Two scales scored 0 (CDAS-R & BSGQ) as no information in the respective papers could be found on item selection. Both CDAS-R & BSGQ scales were developed as extensions to adult versions of the scale. The BSGQ stated the questionnaire is comprehensible to children with language impairments and the CDAS-R stated the questionnaire was tailored for children. However, no evidence was presented in either paper regarding how these conclusions were reached (i.e., whether experts or children were involved).

### Internal Consistency

Most scales (*n* = 6; 75%) achieved the maximum score of three for internal consistency. These were the CAPS, CATS, TOSCA-A, BSGQ, CDAS-R, EVA/ESCA. This indicates that factor analysis had been performed on the scale with an adequate sample size (7* the number of items) and that Cronbach alpha values for the scale or sub-scales fell between 0.70 and 0.95. One measures (TOSCA-A) identified an Omega equivalent to Cronbach’s alpha, a practical alternative for measuring reliability (McDonald 1999), which also fell between these values. The SCS-C scored a two as values fell well below expected standards (i.e., adolescents sample: private = 0.48, public = 0.66; children sample: private = 0.58, public = 0.53). The NASSQ scored one, as a factor analysis was not presented in the paper nor were alpha scores for each dimension of the measure presented, despite an adequate internal consistency score.

### Criterion Validity

One measure obtained the maximum score of three, as the authors included convincing arguments that the “gold standard” was used, and correlations were within the expected range. This was the NASSQ (adolescent version), which correlated with the Children’s Depression Inventory (0.71), Revised Children's Manifest Anxiety Scale (0.73) and the State-Trait Anxiety Inventory (0.74). For the child version, correlations fell just below expected ranges for the Children’s Depression Inventory (0.65), Revised Children's Manifest Anxiety Scale (0.69) and the State-Trait Anxiety Inventory (0.62). A total of four measures (TOSCA-A, CAPS, CDAS-R & BSGQ) obtained a score of two, where although there was evidence for adequate design or method, as well as a convincing argument for using a gold standard, the correlation was less than 0.70. Finally, three measures scored zero—the CATS, SCS-C and EVA/ESCA—as no information on criterion validity could be found.

### Construct Validity

A total of two measures (TOSCA-A & BSGQ) achieved the maximum score of three for this property. This indicted that there was a hypothesis about the relationship between scores on their self-report measure and further measures of theoretically related constructs, with 75% of their findings consistent with stated hypotheses. One measure (CAPS), which included three separated studies in one paper, obtained a score of 1 because a specific hypothesis was formulated for study two, but for study 3 hypotheses were put forward posteriori. The remaining five measures (CATS, NASSQ, CDAS-R, EVA/ESCA & SCS-C) did not offer a clear hypothesis nor any construct validity information and thus scored 0.

### Reproducibility (Agreement)

Reliability agreement (test–retest) was only reported in 4 of the papers (CATS, SCS-C, CAPS and NASSQ). The CATS achieved the maximum score of three, as test–retest validity was assessed and a convincing argument that agreement was acceptable presented (0.79 at a 1-month interval & 0.76 at a 3-month interval). The SCS-C obtained a score of two, because while information on reliability agreement was provided, only test–retest scores for the adolescent sample, and not the children sample, were included (0.30 for the private sub-scale & 0.67 for the public sub-scale at a 6-month interval); and test–retest reliability was well below standard. Two measures (CAPS & NASSQ), although providing vague reference of acceptable levels of reliability agreement, provided no convincing arguments that agreement was acceptable, thus scored one. For the CAPS, at 1-year interval, 0.65 for self-oriented perfectionism & 0.59 for socially prescribed perfectionism were achieved. At 3-year interval, 0.51 for self-oriented perfectionism & 0.35 for socially prescribed perfectionism were achieved. At 5-year interval, 0.40 for self-oriented perfectionism & 0.36 for socially prescribed perfectionism were achieved. For the NASSQ, at 2-week interval 0.96 & 0.78 were achieved for the child and adolescent sample, respectively. The remaining four measures (TOSCA-A, BSGQ, CDAS-R & EVA/ESCA) neither referred to reliability agreements nor absolute measurement error and thus scored zero.

### Interpretability

No measure achieved the highest rating for this property. Three measures achieved a score of one. These were the CAPS, CATS and the NASSQ, as they included descriptive statistics for multiple groups, comparative data on the distribution of scores, information on the relationship of scores to other measures and/or clinical diagnoses. However, none defined the Minimally Important Change (MIC). The CAPS reported the mean and standard deviation for multiple groups including clinical and non-clinical samples in populations from China, Canada, Israeli and Russia. The NASSQ also reported the mean for differing clinical populations (depression and anxious groups), and the CATS reported the mean for a community sample, anxious sample, depressed sample and behaviour disorder sample. The remaining five measures (TOSCA-A, BSGQ, CDAS-R, EVA/ESCA & SCS-S) did not report information on interpretability, scoring zero.

### Cross-Cultural Validity

Only two measures, the CAPS (developed in Canada) and the EVA/ESCA (developed in Brazil) reported cross-cultural validity. The CAPS scored a 2 as there was evidence of forward and back translation in accordance with established practices, but no evidence of pre-testing with children nor adolescents. The remaining measure (EVA/ESCA) scored zero as it included no translation process information.

### Readability

Only one measure, the CAPS, included information on readability. The CAPS used Flesch Kincaid readability standard formulae for reading-level analysis of items and instructions. For this an adequate level was established. Thus, the CAPS scored three. The BSGQ stated that items are comprehensible to children with language impairments, however, no readability score was included in the study. Thus, the BSGQ alongside all other measures obtained a zero, as no information on readability could be found.

### Missing Metrics

No measure provided information on reproducibility reliability (i.e., information on Intraclass Correlation Coefficient (ICC) or weighted Kappa), floor/ceiling effects nor responsiveness. Thus, all eight measures scored zero for these criteria.

### Overall Quality

Overall quality scores are summarised in Table 3. Scores varied from 5 to 12 (*m* = 7.75, median = 8, mode = 5, SD 2.43) out of a total of 27. This is in the expected range with previous systematic reviews, which also revealed that the majority of scales fail to achieve high or very high scores (e.g., Bentley et al. 2019). Of note, in the current systemic review, the CAPS obtained the highest score of 12 while three scales CDAS-R, EVA/ESCA & SCS-S scored the lowest value of 5.

## Discussion

The purpose of this study was to identify measures that investigate negative self-referential emotions in child and adolescent populations and evaluate the psychometric properties of these measures. Eight measures were identified, which formed three distinct categories: (i) the BSGQ, EVA/ESCA, SCS-C and TOSCA-A measured negative self-conscious emotions; (ii) the CATS and NASSQ measured negative self-cognitions; and (ii) the CDAS-R and CAPS measured perfectionism. In this final section of the review, we synthesise evidence from our evaluation and discuss key issues that have arisen.

Of the eight measures, most reported an average of three to four of the eleven psychometric properties. The CAPS examined the most (eight), making it the most psychometrically rigorous measure. The NASSQ examined five, the CATS and TOSCA-A examined four, the BSGQ and SCS-C three, and the CDAS-R and EVA/ESCA provided evidence pertaining to only two of the eleven psychometric properties. No measure provided information on floor or ceiling effects, nor responsiveness. The CAPS was the highest scoring scale (12/27), with an average rating of 7.75 across measures. This suggests that the quality and reporting of psychometric properties in the published scale development papers is limited and potentially of poor quality. This accords with findings of Bentley et al. (2019) who also noted the poor quality of psychometric measures in their review of scales for use with adolescent mental health (i.e., clinical) populations.

The review revealed internal consistency was the most frequently reported psychometric property, with Cronbach alpha coefficients in the acceptable range for all measures but the SCS-C. However, far less attention was paid to other psychometric criteria, such as criterion validity, test–retest reliability and interpretability. Indeed, in this review, these properties were either untested or—more often—of low psychometric quality. Correlations with other measures were reported in five of the eight measures, these being the CAPS, TOSCA-A, NASSQ, BSGQ and CDAS-R. However, only for the NASSQ (adolescent version) did reports of the criterion correlation fall within recommended criteria. In this case, ‘gold standard’ mental health constructs of depression (Children’s Depression Inventory) and anxiety (Revised Children’s Manifest Anxiety Scale and the State-Trait Inventory). The remaining four measures reported weak (*r* < 0.5) and/or statistically non-significant correlations with other mental health or emotional wellbeing constructs. Importantly, information on criterion validity was missing for the CATS, EVA/ESCA and SCS-C. Consequently, further evaluation of how these measures correlate with other psychological constructs is required to add to the utility and validity of these measurement tools.

In explaining these results, it is quite possible that criterion validity was of low psychometric quality (four measures) or not tested (three measures) given that proper investigation of the proposed negative self-directed emotion had not been extensively researched prior to scale development. This includes establishing that children of the youngest ages understood the construct or domain under investigation (e.g., shame, hostility, guilt etc.). A point that we return to later in our discussion of methodological issues.

Considering next test–retest reliability (i.e., agreement), this was reported in four of the eight measures, including the CAPS, CATS, NASQ and SCS-C. All four evaluations of test–retest reliability reported Pearson’s or Spearman’s correlations, rather than Kappa or ICCs as recommended by Terwee et al. (2007). Here, test–retest reliability was generally inadequate, with only the CATS scoring the maximum of three for this property and providing a convincing argument that the correlation coefficient was acceptable after both 1- and 3-month intervals. The SCS-C, NASQ and CAPS all showed evidence of psychometric limitations for this property; although all of the latter provided a test for reliability, the correlation reported for the SCS-C was low and neither the NASQ nor CAPS provided an explanation of whether agreement observed was acceptable or not. Disappointingly, no test for reliability was conducted for the TOSCA-A, BSGQ, CDAS-R nor the EVA/ESCA. This limits the efficacy of these measures to be used in the assessment of emotional wellbeing, particularly if an individual or group are wanting to use the measure as a tool to investigate intervention effectiveness over a period of time. For those measures where test-re-test was reported and reliability found to be inadequate, one suggestion here is that the construct under investigation is potentially not stable, or that the measure is not sufficiently well developed to measure the construct reliably. A point we have made earlier and return to below.

Interpretability was also poorly reported, with only the CAPS, CATs and NASQ including data for non-clinical/community samples as well as clinical samples. Additionally, except for the CAPs, there appeared to be little analysis of the applicability of the measures to different groups of children and adolescents, including populations from different ethnic groups and/or cultures. The CAPS was the only measure to include samples from other countries (e.g., China, Israel & Russia), suggesting it may be useful when exploring negative self-referential emotions across different populations, or the international utility of specific interventions.

Additionally, with the exception of the EVA/ESCA, all included measures were developed and validated in westernised cultures/populations. Namely, American/Canadian (CAPS, CDAS-R & NASSQ), European (BSGQ), Australian (CATS & TOSCA) or UK (SCS-C) populations. Thus, for these measures (bar the CAPS), transferability to different populations cannot be automatically assumed. It is also worth noting that whilst the EVA/ESCA was translated to English (from either Spanish or Portuguese), no information pertaining to the translation process was reported. Again, this limits the international utility of the reported measures, which would allow researchers, practitioners and policy makers to evaluate school- or community-based emotional and mental wellbeing interventions in a wider range of populations.

Aside from reports of psychometric properties, several methodological issues were observed, especially concerning the age-appropriateness of the measures for younger children. There appeared to be little consideration of the comprehension of questions for young people in the measures reported. This is a salient issue for the BSGQ, CATS, CDAS-R & TOSCA-A, as all are claimed to be suitable for adolescent *and* child samples. Importantly, research examining emotional word comprehension demonstrates that complex emotion vocabulary develops dramatically during childhood, doubling in size every 2 years between the ages of 8 and 11 (Baron-Cohen et al. 2010). Therefore, a measure suitable for 15-year olds may not be suitable for 8-year olds. Measures that are too complex can also result in lower data quality and missing data (De Leeuw 2011). Yet only the CAPS included readability metrics. Given the arguments of Baron-Cohen et al. more consideration of readability is required by researchers when designing emotional measures for child and adolescent populations to ensure they are developmentally sensitive. Certainly, if a child does not understand the measure items, then this would not only affect content and construct validity, but also test-rest reliability.

This review further demonstrated that child and adolescent involvement in the item generation process was minimal. The CATS was the only measure to involve in-depth interviews with both children and adolescents, as well as the consultation of experts during the initial item pool generation. Additionally, while the EVA/ESCA and NASSQ included children/adolescents in item generation, experts were not involved. Finally, there was no evidence of any involvement of children nor adolescents in critical stages of the item development process for the CAPS, TOSCA-A, CDAS-R, SCS-C, BSGQ. This issue is of particular importance for the BSGQ & CDAS-R, as they are scales adapted from adult versions. As their original development was with adult populations, a lack of involvement of young people is particularly problematic. For example, De Leeuw (2011) highlights that involvement of children and/or adolescents in the early stages of the development of an emotional wellbeing-measure is necessary to ensure that the measure is not only age-appropriate, but also to ensure child/adolescent understanding of the construct under investigation. Added to this, in their review of emotion development in children, Hoemann et al. (2019) evidence that even by the age of 9, children’s ability to label even traditional categorical emotions (e.g., disgust) is not fully resolved, and when considering emotions that are self-referential, these may very much align with the expectations and patterns of a child’s culture. As an example, they note how in individualistic cultures, an award in school may be associated with a developing construct of pride, whereas in collectivist cultures this very same experience may lead to the resulting emotional learning being more akin to ‘respectful deference or even embarrassment’. For these reasons, we argue that in the development of measures suitable for use in youth (and especially younger-aged children), it is essential that the relevant populations serve as experts in the scale development process in order for construct validity to be met.

A further observation noteworthy of mention concerns the length of measures and response style adopted, which may limit the use of certain measures included in this review. As highlighted by Deighton et al. (2014), longer measures are less likely to be selected with younger populations. The CATS consisting of 40-items is a relatively long measure. This potentially poses problems of cognitive load, attention span and working memory, especially in child populations. As such, researchers and clinicians may find it useful to select relevant sub-scales from this measure, as appropriate. Furthermore, all studies reported in this systematic review used Likert scales, ranging from three-point (BSGQ) to seven-point (SCS-C) scales. Chambers and Johnston (2002) have tested children aged 5–12 years and found that younger children tend to endorse responses at the extreme end of scales when presented with items based on a Likert scale. It is therefore worth noting that the BSGQ may result in limited variability in the data derived given its lack of scale points. This could possibly lead to issues of insensitivity to change over time and/or floor/ceiling effects, if used to measure change.

The lack of evidence on sensitivity to change is moreover of particularly concern if researchers, educators/teachers and/or practitioners, use the reviewed measures to monitor the effectiveness of wellbeing interventions over time, rather than for cross-sectional purposes. The ability of a measure to detect changes over time is extremely important. Policy makers, researchers and practitioners are expanding the provision of school- and community-based mental health interventions and services, and increasingly using emotional wellbeing measures to evaluate these interventions. We recommend future studies should place a greater focus on investigating the sensitivity and responsiveness to change of emotional measures. This can be achieved by conducting longitudinal scale development/self-report studies with child and adolescent populations to investigate change over time in respect to the (negative) self-referential emotion in question.

Finally, although we located a total of 98 negative emotional measures for children and/or adolescents, only 20% incorporated an item examining self-referential emotions. Most measures examined generic emotions, such as ‘frequency of feeling a negative emotion’ in various contexts, including toward family, friends, when at home or when in school, rather than that emotion toward the self. In fact, no measure of aggression, anger, fear, disgust, pessimism, loneliness, narcissism, hopelessness, stress, worry or sadness, that we found, included a self-referential item. Additionally, no specific measures of self-criticism for child or adolescent populations were found. Considering the prevalence of such measures in the adult literature (Rose and Rimes 2018), and the argument that self-criticism is a major source of vulnerability to psychopathology (Gilbert and Irons 2005; Kannan and Levitt 2013; McIntyre et al. 2018; Zuroff et al. 2005), this clearly is important to address. Albeit, prior to such scale development, proper investigation that the construct is applicable in child populations should be undertaken. For example, qualitative data collection and analysis, combined with adopting a critical realist approach would allow for greater exploration of (negative) self-referential emotions in children/youth, whilst also acknowledging the socially constructed nature of worldly knowledge (Archer et al. 2013; see also Hoemann et al. 2019).

### Limitations

Our scale recommendations and considerations are, however, not without limitation. The primary limitation concerns the stringent inclusion and exclusion criteria adopted. This included, where possible, original scale development papers, which reported psychometric properties only. Therefore, additional studies on these measures that further illuminate their psychometric properties may have been missed. Furthermore, all measures pertaining to specific psychological disorders based on clinical diagnoses, the DSM or broad measures of mental health were excluded; because the aim of this review was to identify measures of negative self-referential emotions that can be used within general populations. This was driven by issues of resource/service challenge, and potential floor/ceiling effects when using clinical diagnostic measures with non-clinical populations (Orth and Wyk 2020). We do, however, recognise that many of these clinical measures are psychometrically comprehensive (but see here Bentley et al. 2019) and of practical use in other settings or with specific groups; especially those with a mental-health diagnosis.

Finally, this systematic review was confined to examining only negative self-referential measures of emotion. Our rationale for this was that negative self-referential emotions portend vulnerability to a range of mental-health disorders/psychopathologies. In doing so, however, it is quite possible that some broader more generic measures of wellbeing were excluded, including those that present a blend of positive and negative emotions. For example, the Rosenberg self-esteem scale (Rosenberg 1965) is a ten-item measure that includes two negative self-referential emotions that map onto failure. Nonetheless, as it is not a specific measure of negative self-referential emotions (i.e., it measures positive feelings about the self), it did not meet our search criteria. Indeed, Pollard and Lee (2003) urge caution when using traditional presumed global indicators of wellbeing (specifically citing self-esteem and depression) observing that *‘wellbeing is more than a sole indicator in a single domain’* (p. 67). Certainly, the link between self-referential emotions and vulnerability to mental disorders, thus far mainly researched in adult populations, highlights this very point.

## Conclusion

To conclude, self-reported and self-referential measures of negative emotion may not only be essential in establishing estimates of wellbeing in child and adolescent samples, but also in the evaluation of emotional wellbeing intervention efficacy, especially in general (i.e., predominantly non-clinical) populations. In this review, 98 negative measures of wellbeing were identified for use with young people, but only eight measured a negative self-referential emotion. Of these, all had significant shortcomings in terms of psychometric evaluation; although notably the CAPS was evaluated as the most rigorous in psychometric quality, followed by the CATS.

Our review additionally raised concerns with respect to: appropriateness (e.g., readability and proper validity checks); the extent to which the eight reported measures are sensitive to change over time; and that adequate exploration of the said negative self-referential emotional construct had been undertaken prior to scale development. This indicates a clear need for further evaluation of such measures, including more focus on content- and criterion-related validity. This stated, the eight measures identified in this review provide a substantial array of tools for mental health researchers. Indeed, in providing a comprehensive review of each measure this review will help researchers to make informed decisions about which tool or tools to use when investigating negative self-referential emotions to either support understanding of child and adolescent development (especially in the context of the self); and/or evaluate the efficacy of emotional wellbeing interventions with school- and community-based child and adolescent populations.

## Supplementary Information

Below is the link to the electronic supplementary material.

Electronic supplementary material 1 (DOCX 56 kb)
