# Supplementary material for: A Systematic Review of Self-Report Measures of Negative Self-Referential Emotions Developed for Non-Clinical Child and Adolescent Samples
Source: Clin Child Fam Psychol Rev. 2021 Feb 5;24(2):224–43. doi: 10.1007/s10567-020-00339-9 (PMC7862045; doi:10.1007/s10567-020-00339-9)
Supplement: Supplementary file 1 — Electronic supplementary material 1 (DOCX 56 kb) [file 10567_2020_339_MOESM1_ESM.docx]

**Supplementary Table:** Measures excluded and reasons for their exclusion

| **Domain** | **Measure** | **Abbreviation** | **Reason for Exclusion** |
| --- | --- | --- | --- |
|  | **Diagnostic Measures (21)** | | |
| Depression (8) | Child Depression Inventory  (Kovacs,1992) | CDI | Based on DSM |
|  | Reynolds Adolescents Depression Scale (Reynolds, 2002) | RADS | Based on DSM |
|  | The Multidimensional Child and Adolescent Depression Scale (Abdel-Khalek, 2003). | MCADS | Based on DSM |
|  | Screening Test for Depression (Hegerl, 2020) | STD | Based on DSM |
|  | The Epidemiologic Studies Depression Scale (Radloff, 1991) | CES-DC | Based on DSM |
|  | Moods and Feelings Questionnaire (Costello and Angold, 1988)  Children Depression Experiences Questionnaire (Abela et al., 2012) | MFQ  CDEQ | Based on DSM  Based on DSM |
|  | Depression Experiences Questionnaire-Adolescents (Blatt et al., 1992) | DEQ-A | Based on DSM |
| Anxiety (7) | State-Trait Anxiety Inventory (Spielberger, 1973) | STAI | Based on DSM |
|  | Junior Manifest Anxiety Scale (Joshi, 1974) | N/A | Based on DSM |
|  | The Multidimensional Anxiety for Children (March et al., 1997) | MASC | Based on DSM |
|  | Screen for Child Anxiety Related Emotional Disorder (Birmaher et al., 1999) | SCARED | Based on DSM |
|  | State Trait Anxiety Inventory for Children (Spielberger, 1973) | STAIC | Based on DSM |
|  | Youth Anxiety Measure for DSM-5 (Muris et al., 2017) | YAM-5 | Based on DSM |
|  | Spence Children Anxiety Scale (Spence, 1997) | SCAS | Based on DSM |
| Multi-dimensional Depression and Anxiety (4) | Multifactorial Scale of Anxiety (Fenz & Epstein, 1965) | N/A | Based on DSM |
|  | Depression Anxiety Stress Scale for adolescents (Lovibond & Lovibond, 1995) | DASS | Based on DSM |
|  | Revised Children Anxiety and Depression Scale (Chorpita et al., 2000) | RCADS | Based on DSM |
|  | Beck Youth Inventories of Emotional and Social Impairment (Beck et al., 2005) | BYI | Based on DSM |
| Obsessive Compulsive Disorder (2) | The Leyton Obsessional Inventory-Child Version (Berg, Rapoport, & Flament, 1986) | LOI-CV | Based on DSM |
|  | Obsessive Beliefs Questionnaire- Child version (Coles et al., 2010) | OBQ-CV | Based on DSM |
|  | **Specific Indicators of Negative Self-referential Emotional Measures (34)** | | |
| Anger (10) | DSM-5 Level 2 Anger scale Children and adolescent Validation (Sapmaz et al. 2017) | Level 2 | Based on DSM |
|  | State-Trait Anger Expression Inventory for children and adolescents (Del Barrio, Aluja, & Spielberger, 2004) | STAXI-CA | No SR emotional items |
|  | Paediatric Anger Expression Scale (Hagglund et al. 1994) | PAES | No SR emotional items |
|  | Affective Reactivity Index (Stringaris et al. 2012) | ARI | No SR emotional items |
|  | Anger Expression Scale for children (Steele et al. 2009) | AESC | No SR emotional items |
|  | Anger Regulation and Expression Scale (Lampert, 2014). | N/A | Dissertation |
|  | Anger Inventory (Alcázar-Olán et al. 2012) | N/A | Items not in English |
|  | Children lab game anger (Hubbard, 2005) | N/A | Behavioral measure |
|  | Anger Behavioral observation (Rohlf & Krahe, 2015) | N/A | Behavioral measure |
|  | Holistic Anger Rating Scale (Mani et al. 2018) | N/A | No SR emotional items |
| Aggression (3) | Olweus Aggression Inventory (Ekblad, & Olweus,1986) | N/A | No SR emotional items |
|  | Aggression Questionnaire- Preadolescents and adolescents (Santisteban & Alvardo, 2009) | AQ-PA | Items not in English |
|  | The Aggression Questionnaire (Buss & Perry, 1992) | N/A | No SR emotional items |
| Fear (4) | Fear Experiences Questionnaire for adolescents (Gullone, King & Ollendick, 2000). | FEQ | No SR emotional items |
|  | Revised Children Fear Survey Schedule (Ollendick, 1983). | FSSC-R | No SR emotional items |
|  | The Koala Fear Test (Muris et al., 2003)  Children Fear Test (Crohn, 1997) | N/A  N/A | No SR emotional items  Dissertation |
| Disgust (2) | Child Disgust Scale (Viar-Paxton et al. 2015) | CDS | No SR emotional items |
|  | Disgust Emotion scale for Children (Muris et al. 2012) | DES-C | No SR emotional items |
| Perfectionism (2) | Perfectionistic Self-presentation scale- Junior (Hewitt et al. 2011) | PSPS-JR | No SR emotional items |
|  | Almost Perfect Scale-Revised (Adults- Slaney et al. 2001) | APS-R | No SR emotional items |
| Pessimism (2) | Youth Life Orientation Test (Ey et al. 2005) | YLOT | No SR emotional items |
|  | Parent rated Life Orientation Test for children (Lemola et al. 2010) | PLOT | Parent rated |
| Loneliness (4) | Loneliness and Aloneness Scale for children (Marcoen, Goossens, & Caes, 1987) | LACA | No SR emotional items |
|  | De Jong Gierveld Loneliness Scale (Grygiel, Humenny & Rębisz, 2019) | DJGLS | No SR emotional items |
|  | Children Loneliness Scale (Asher, Hymel & Renshaw, 1984) | CLS | No SR emotional items |
|  | Perth A-Loneliness Scale (Houghton, Hattie, Wood, Carroll, Martin & Tan, 2014) | PALS | No SR emotional items |
| Narcissism (2) | Childhood Narcissism Scale (Sander et al. 2008) | CNS | No SR emotional items |
|  | Narcissistic Personality Questionnaire- children (Ang & Yusof, 2006) | NPQC | No SR emotional items |
| Hopelessness (1) | Hopelessness Scale for Children (Spirito et al. 1988) | HSC | No SR emotional items |
| Stress (2) | The perceived Stress scale for children (White, 2014) | PSS-C | No SR emotional items |
|  | The Daily Life Stressors Scale (Kearney, Drabman & Beasley, 1993) | DLSS | No SR emotional items |
| Worry (1) | Penn State Worry Questionnaire for Children (Chorpita et al. 1997) | PSWQ | No SR emotional items |
| Sadness (1) | Children’s Sadness Management Scale (Zeman, Shipman & Penze-Clyve (2001) | N/A | No SR emotional items |
|  | **Negative Self-conscious Emotion Measures (6)** | | |
| Self-conscious emotions (2) | Self-conscious Emotions Questionnaire for children (Haimowitz, 1996) | SCEQC | Dissertation |
|  | Guilt and Shame Questionnaire—for Adolescents of Parents with a Mental Illness (Bosch et al. 2020) | GSQ-APMI | Specific population |
| Shame (2) | Shame Questionnaire (Mcleod, 2003) | N/A | Dissertation |
|  | Adolescents Shame Proneness Scale (Simonds et al. 2016) | ASPS | No SR emotional items |
| Guilt (1) | Interpersonal Guilt Questionnaire (Mulherin, 1998) | IGQ-Adol | Dissertation |
| Moral Emotions (1) | Moral Development (Smith, 1995) | N/A | Dissertation |
|  | **Non-self-directed emotional measures (22)** | | |
|  |  |  |  |
| Social Anxiety measures (3) | Lebowitz Social Anxiety Scale for Children and Adolescents, self-report version (Rodríguez et al. 2009) | LSAS-CA | Not a SR emotional domain |
|  | Social Anxiety Scale for children (La Greca et al. 1988) | SASC | Not a SR emotional domain |
|  | Social Anxiety Questionnaire for Children (SAQ-C; Caballo et al. 2016) | SAQ-C | Not a SR emotional domain |
| Quality of Life (1) | Quality of Life Scale Children (Thompson et al. 2014) | QOL-C | Not a SR emotional domain |
| Life Satisfaction (4) | Satisfaction with Life Scale adapted for Children (Gadermann, Schonert-Reichl, & Zumbo, 2010) | SWLS-C | Not a SR emotional domain |
|  | Multidimensional Students' Life Satisfaction Scale (Huebner, 1991) | MSLSS | Not a SR emotional domain |
|  | The Personal Wellbeing Index Scale (Tomyn, Fuller-Tyszkiewicz & Cummins, 2013) | PWI-SC | Not a SR emotional domain |
| Self-concept (2) | The Piers-Harris Self-concept scale (Piers & Herzberg, 2002) | N/A | Not a SR emotional domain |
|  | Harters Self-perception scale for children (Harter, 1982) | N/A | Not a SR emotional domain |
| Anxiety Sensitivity (3) | Childhood Anxiety Sensitivity Index (Silverman et al. 1991) | CASI | Not a SR emotional domain |
|  | Anxiety Sensitivity Index for Children (Laurent et al. 1999) | ASIC | Not a SR emotional domain |
|  | Childhood Anxiety Sensitivity Index: Behavioural validation (Rabian,, Embry, and MacIntyre, 1999) | N/A | Not a SR emotional domain/ Behavioural measure |
| Emotion Regulation (4) | How I feel (Walden, Harris & Catron, 2003) | HIF | Not a SR emotional domain |
|  | Emotional Awareness Questionnaire (Rieffe et al. 2007) | EAQ | Not a SR emotional domain |
|  | Emotion Regulation Questionnaire- Children and adolescents (Gullone & Taffe, 2012) | ERQ-CA | Not a SR emotional domain |
|  | Difficulties in Emotion Regulation-18 (Victor, & Klonsky, 2016). | DERS-18 | Not a SR emotional domain |
|  | FEEL-KJ (Cracco, Van Durme, & Braet, 2015) | N/A | Not a SR emotional domain |
| Positive/Negative Affect (2) | Positive and Negative Affect Schedule for children  Laurent et al., 1999) | PANAS-C | Not a SR emotional domain |
|  | Positive and Negative Affect Schedule—Expanded Form (PANAS–X; Watson & Clark, 1994). | PANAS-X | Not a SR emotional domain |
| Social Emotional (1) | Strong Souls (Thomas et al. 2010) | N/A | Not a SR emotional domain |
| Coping Styles (2) | Children’s Response Style Questionnaire (Lo et al. 2017) | CRSQ | Not a SR emotional domain |
|  | KidCope (Spirito, Stark & Williams, 1988) | KidCope | Not a SR emotional domain |
| **Multi-dimensional measures of wellbeing (7)** | | | |
|  | Kid screen 27-short version (Ravens-Siebere et al., 2010) | KIDSCREEN | Multi-dimensional measure |
| General multi-dimensional measures | Strengths and Difficulties Questionnaire (Goodman, 2001) | SDQ | Multi-dimensional measure |
|  | Mental Outcome Survey-SF (McHorney, Ware, and Raczek., 1993) | MOS-SF | Multi-dimensional measure |
|  | Kidscreen 27 (Herdman et al., 2002) | N/A | Multi-dimensional measure |
|  | Warwick Edinburgh Mental Wellbeing Scale (Tennant et al. 2007) | WEMWBS | Multi-dimensional measure |
|  | KINDL (Ravens-Sieberer & Bullinger, 1998)  Children’s Moods, Fears and Worries Questionnaire (Bayer, Sanson & Hemphill, 2006) | KINDL  CMFWQ | Multi-dimensional measure / SR items is positive emotion  Multi-dimensional measure / pre-school children |

SR= Self-referential, DSM=Diagnostic and Statistical Manual.

**Supplementary Table References**

Abdel-Khalek, A. M. (2003). The Multidimensional Child and Adolescent Depression Scale: Psychometric

Properties. *Psychological Reports, 93*(2), 544–560.

Abela, J. R., Fishman, M. B., Cohen, J. R., & Young, J. F. (2012). Personality predispositions to depression in

children of affectively-ill parents: The buffering role of self-esteem. *Journal of Clinical Child & Adolescent Psychology, 41*(4), 391-401.

Alcázar-Olán, R. J., Deffenbacher, J. L., Pérez, V. R., & Pool Cibrián, W. J. (2012). Developing an Inventory to

Measure Anger in Mexican Children. *Desarrollo de Un Inventario Para La Medición de La Ira En Niños Mexicanos, 21*(2), 303

Ang, R. P., & Yusof, N. (2006). Development and Initial Validation of the Narcissistic Personality

Questionnaire for Children: A preliminary investigation using school‐based Asian samples*. Educational Psychology, 26*(1), 1.

Asher, Hymel, & Renshaw (1984) Loneliness in children. *Child Development, 55,* 1456-1464.

Bayer, J. K., Sanson, A. V., & Hemphill, S. A. (2006). Children’s Moods, Fears, and Worries: Development of

an Early Childhood Parent Questionnaire. *Journal of Emotional and Behavioral Disorders, 14*(1), 41–49.

Beck, A., Beck, J., Jolly, J., and Steer, R. (2005) *Beck Youth Inventories™ - Second Edition For Children and*

*Adolescents (BYI-II)*, Pearson.

Berg, C. J., Rapoport, J. L., & Flament, M. (1986). The Leyton obsessional inventory-child version. *Journal of*

*the American Academy of Child Psychiatry, 25*(1), 84-91.

Birmaher, B., Brent, D. A., Chiappetta, L., Bridge, J., Monga, S., & Baugher, M. (1999). Psychometric

properties of the Screen for Child Anxiety Related Emotional Disorders (SCARED): A replication study. *Journal of the American Academy of Child and Adolescent Psychiatry, 38*(10), 1230–6.

Blatt, S J., et al. (1992) Psychometric Properties of the Depressive Experiences Questionnaire for Adolescents.

*Journal of Personality Assessment*, *59* (1), 82-98.

Bosch, A., van de Ven, M. & van Doesum, K. (2020) Development and Validation of the Guilt and Shame

Questionnaire for Adolescents of Parents with a Mental Illness (GSQ-APMI). *Journal of Child and Family Studies 29,* 1147–1158 (2020).

Buss, A.H., & Perry, M. (1992). The Aggression Questionnaire. *Journal of Personality and Social Psychology,*

*63,* 452-459.

Caballo, V. E., Salazar, I. C., Arias, B., Calderero, M., Irurtia, M. J., Ollendick, T. H., & CISO-A Research

Team. (2016). The Social Anxiety Questionnaire for Children: Cross-cultural assessment with a new self-report measure. *Journal of Psychopathology and Behavioral Assessment, 38*(4), 695–709.

Chorpita, B. F., Tracey, S. A., Brown, T. A., Collica, T. J., & Barlow, D. H. (1997). Assessment of worry in

children and adolescents: An adaptation of the Penn State Worry Questionnaire. *Behaviour Research and Therapy, 35*(6), 569-58

Chorpita, B. F., Yim, L., Moffitt, C., Umemoto, L. A., & Francis, S. E. (2000). Assessment of symptoms of

DSM-IV anxiety and depression in children: A revised child anxiety and depression scale. *Behaviour research and therapy, 38*(8), 835-855.

Coles ME, Wolters LH, Sochting I, de Haan E, Pietrefesa AS, Whiteside SP. (2010) Development and initial

validation of the obsessive belief questionnaire-child version (OBQ-CV). *Depression and Anxiety, 27*(10), 982-991.

Costello, E. J., & Angold, A. (1988). Scales to Assess Child and Adolescent Depression: Checklists, Screens,

and Nets. *Journal of the American Academy of Child and Adolescent Psychiatry*, *27*(6), 726-737

Cracco, E., Van Durme, K., & Braet, C. (2015). Validation of the FEEL-KJ: An Instrument to Measure Emotion

Regulation Strategies in Children and Adolescents. *PloS one*, *10*(9), e0137080.

Crohn, T. M. (1997). *The psychometric properties of the children’s fear test*. Dissertation Abstracts

International: Section B: The Sciences and Engineering. ProQuest Information & Learning, US.

del Barrio, V., Aluja, A., & Spielberger, C. (2004). Anger assessment with the STAXI-CA: psychometric

properties of a new instrument for children and adolescents. *Personality & Individual Differences, 37*(2), 227–244.

Ekblad, S., & Olweus, D. (1986). Applicability of Olweus’ Aggression Inventory in a Sample of Chinese

Primary School Children. *Aggressive Behavior, 12*(5), 315.

Ey, S., Hadley, W., Allen, N., Palmer, S., Klosky, J., Deptula, D., … Cohan, R. (2005). A new measure of

children’s optimism and pessimism: The youth life orientation test. *Journal of Child Psychology and Psychiatry,* 46(5), 548-558.

Fenz, W. D., & Epstein, S. (1965). Manifest anxiety: Unifactorial or multifactorial composition? *Perceptual and*

*Motor Skills, 20*(3), 773-780.

Gadermann, A. M., Schonert-Reichl, K. A., & Zumbo, B. D. (2010). Investigating validity evidence of the

Satisfaction with Life Scale adapted for Children. Social Indicators Research, *96*, 229-247.

Goodman. R. (2001). Psychometric properties of the strengths and difficulties questionnaire. *Journal of the*

*American Academy of Child and Adolescent Psychiatry, 40*(11), 1337-1345

Grygiel, P., Humenny, G., & Rębisz, S. (2019). Using the De Jong Gierveld Loneliness Scale With Early

Adolescents: Factor Structure, Reliability, Stability, and External Validity. *Assessment*, *26*(2), 151–165.

Gullone, E., & Taffe, J. (2012). The Emotion Regulation Questionnaire for Children and Adolescents (ERQ–

CA): A psychometric evaluation. *Psychological Assessment, 24*(2), 409–417.

Gullone, E., King, N. J., & Ollendick, T. H. (2000). The development and psychometric evaluation of the Fear

Experiences Questionnaire: an attempt to disentangle the fear and anxiety constructs. *Clinical Psychology & Psychotherapy, 7*(1), 61-75.

Hagglund, K. J., Clay, D. L., Frank, R. G., Beck, N. C., Kashani, J. H., Hewett, J., … Cassidy, J. T. (1994).

Assessing anger expression in children and adolescents. *Journal Of Pediatric Psychology, 19*(3), 291–304.

Harter, S. (1982). The Perceived Competence Scale for Children. *Child Development, 53*(1), 87.

Herdman, M., Rajmil, L., Ravens-Sieberer, U., Bullinger, M., Power, M., & Alonso, J. (2002). Expert consensus

in the development of a European health-related quality of life measure for children and adolescents: a Delphi study. *Acta Paediatrica, 91*(12), 1385-1390.

Hewitt, P. L., Blasberg, J. S., Besser, A., Flett, G. L., Sherry, S. B., Caelian, C., … Birch, S. (2011).

Perfectionistic Self-Presentation in Children and Adolescents: Development and Validation of the Perfectionistic Self-Presentation Scale-Junior Form. *Psychological Assessment, 23*(1), 125–142.

Laurent, j., Schmidt, B., Catanzaro, S., Joiner, T. & Kelley, A. (1998) Factor Structure of a Measure of Anxiety

Sensitivity in Children. *Journal of Anxiety Disorders*, 12(4), , 307-331.

Haimowitz, B. R. (1996, October). *The assessment of shame and guilt in elementary school children.*

Dissertation Abstracts International Section A: Humanities and Social Sciences. ProQuest Information & Learning, US.

Hegerl, A. (2020) Screening Test for Depression. <https://www.deutsche-depressionshilfe.de/start>

Houghton, S., Hattie, J., Wood, L., Carroll, A., Martin, K., & Tan, C. (2014). Conceptualising loneliness in

adolescents: Development and validation of a self-report instrument. *Child Psychiatry and Human Development, 45*(5), 604–616

Hubbard, J. A. (2005). Eliciting and Measuring Children’s Anger in the Context of Their Peer Interactions:

Ethical Consideration Practical Guidelines. *Ethics & Behavior, 15*(3), 247–258.

Huebner, E.S. (1991). Initial development of the Students’ Life Satisfaction Scale. *School Psychology*

*International, 12*, 231-243

Joshi, R. T. (1974). Field-dependence, anxiety, and personality. *Perceptual and Motor Skills, 38*, 1328

Kearney, C. A., Drabman, R. S., & Beasley, J. F. (1993). The trials of childhood: The development, reliability,

and validity of the Daily Life Stressors Scale. *Journal of Child and Family Studies, 2*(4), 371–

388.

Kovacs M. (1992) *The Children’s Depression Inventory Manual*. New York, NY: Multi-Health Systems.

La Greca, A. M., Dandes, S. K., Wick, P., Shaw, K., & Stone, W. L. (1988). Development of the Social Anxiety

Scale for Children: Reliability and concurrent validity. *Journal of Clinical Child Psychology*, 17(1), 84-91.

Lampert, A. (2014). *An experimental test of the anger impression scales of the ares*. Dissertation Abstracts

International: Section B: The Sciences and Engineering. ProQuest Information & Learning, US.

Laurent, J., Catanzaro, S. J., Joiner, T. E., Jr., Rudolph, K. D., Potter, K. I., Lambert, S., . . . Gathright, T.

(1999). A measure of positive and negative affect for children: Scale development and preliminary validation. *Psychological Assessment,* *11*(3), 326-338.

Lemola, S., Räikkönen, K., Matthews, K. A., Scheier, M. F., Heinonen, K., Pesonen, A.-K., … Lahti, J. (2010).

A new measure for dispositional optimism and pessimism in young children. *European Journal of Personality, 24*(1), 71–84.

Lo, B., Zhao, Y., Ho, Y. C., & Au, T. K. (2017). Psychometric properties of the Children's Response Styles

Questionnaire in a Hong Kong Chinese community sample. *Health and quality of life outcomes*, *15*(1), 198.

Lovibond, P. F., & Lovibond, S. H. (1995). The structure of negative emotional states: Comparison of the

Depression Anxiety Stress Scales (DASS) with the Beck Depression and Anxiety Inventories. *Behaviour Research and Therapy, 3*3(3), 335–343.

Mani, T.L. & Sharma, Manoj & Omkar, S.N. & Nagendra, H.R.. (2018). Holistic assessment of anger in

adolescents – Development of a rating scale. *Journal of Ayurveda and Integrative Medicine*. 9, 195-200.

March, J. S., Parker, J. D., Sullivan, K., Stallings, P., & Conners, C. K. (1997). The Multidimensional Anxiety

Scale for Children (MASC): factor structure, reliability, and validity. *Journal of the American academy of child & adolescent psychiatry, 36*(4), 554-565.

Marcoen, A., Goossens, L., & Caes, P. (1987). Loneliness in pre through late adolescence: Exploring the

contributions of a multidimensional approach. *Journal of Youth and Adolescence, 16*, 561–577.

McHorney, C. A., Ware Jr, J. E., & Raczek, A. E. (1993). The MOS 36-Item Short-Form Health Survey (SF-

36): II. Psychometric and clinical tests of validity in measuring physical and mental health constructs. *Medical care*, *31*(3) 247-263.

Mcleod, L. D. (2003). *The development and initial validation of the domains of shame questionnaire.*

Dissertation Abstracts International: Section B: The Sciences and Engineering.

Mulherin, K. A. (1998). *Reliability and validity for an adolescent version of the interpersonal guilt*

*questionnaire. Dissertation Abstracts International*: Section B: The Sciences and Engineering. ProQuest Information & Learning, US.

Muris, P., Huijding, J., Mayer, B., Langkamp, M., Reyhan, E., & Olatunji, B. (2012). Assessment of Disgust

Sensitivity in Children With an Age-Downward Version of the Disgust Emotion Scale. *Behavior Therapy, 43*(4), 876–886.

Muris, P., Meesters, C., Mayer, B., Bogie, N., Luijten, M., Geebelen, E., Smit, C. (2003). The Koala Fear

Questionnaire: A standardized self-report scale for assessing fears and fearfulness in pre-school and p rimary school children. *Behaviour Research and Therapy, 41*(5), 597–617.

Muris, P., Simon, E., Lijphart, H., Bos, A., Hale, W. III, Schmeitz, K., & International Child and Adolescent

Anxiety Assessment Expert Group (ICAAAEG). (2017). The Youth Anxiety Measure for DSM-5 (YAM-5): Development and first psychometric evidence of a new scale for assessing anxiety disorders symptoms of children and adolescents. *Child Psychiatry and Human Development, 48*(1), 1–17.

Ollendick, T. H. (1983). Reliability and validity of the Revised Fear Survey Schedule for Children (FSSC-R).

*Behaviour Research and Therapy*, *21*(6), 685–692.

Piers, E. V., & Herzberg, D. S. (2002). *Piers-Harris Children’s Self-Concept Scale-Second Edition Manual.*

Western Psychological Services, Los Angeles, Ca.

Radloff, L. S. (1991). The use of the Center for Epidemiologic Studies Depression Scale in adolescents and

young adults. *Journal of youth and adolescence, 20*(2), 149-166.

Rabian, B., Embry, L., & MacIntyre, D. (1999). Behavioral validation of the Childhood Anxiety Sensitivity

Index in children. *Journal of Clinical Child Psychology, 28*(1), 105-112.

Ravens-Sieberer, U. & Bullinger, M. (1998). Assessing health related quality of life in chronically ill children

with the German KINDL: first psychometric and content-analytical results. Quality of Life Research, 4(7), 399-407

Ravens-Sieberer, U., Erhart, M., Rajmil, L., Herdman, M., Auquier, P., Bruil, J., Power, M., Duer, W., Abel, T.,

Czemy, L., Mazur, J., Czimbalmos, A., Tountas, Y., Hagquist, C., & Kilroe, J. (2010). Reliability, construct and criterion validity of the KIDSCREEN-10 score: a short measure for children and adolescents' well-being and health-related quality of life. *Quality of Life Research, 19*(10), 1487-1500.

Reynolds, W. M. (2002). *Reynolds Adolescent Depression Scale– Second Edition: Professional manual*.

Odessa, FL: Psychological Assessment Resources

Rieffe, C., Meerum Terwogt, M., Petrides, K.V., Cowan, C., Miers, A.C., Tolland, A. (2007). Psychometric

properties of the Emotion Awareness Questionnaire for children. *Personality and Individual Differences, 43,* 95-105.

Rodríguez, José & Sánchez-García, Raquel & López-Pina, J.A.. (2009). The Liebowitz Social Anxiety Scale for

Children and Adolescents. *Psicothema, 21*, 486-91.

Rohlf, H. L., & Krahé, B. (2015). Assessing anger regulation in middle childhood: development and validation

of a behavioral observation measure. *Frontiers in psychology*, *6*, 453.

Santisteban, C., & Alvarado, J. M. (2009). The Aggression Questionnaire for Spanish preadolescents and

adolescents: AQ-PA. *The Spanish Journal Of Psychology, 12*(1), 320–326.

Simonds, L. M., John, M., Fife-Schaw, C., Willis, S., Taylor, H., Hand, H., … Winton, H. (2016). Development

and validation of the Adolescent Shame-Proneness Scale. *Psychological Assessment, 28*(5), 549–562.

Slaney, R. B., Rice, K. G., Mobley, M., Trippi, J., & Ashby, J. S. (2001). The Revised Almost Perfect Scale.

*Measurement and Evaluation in Counseling and Development, 34,* 130–145.

Smith, R. S. (1995). *Toward an objective measure of moral development for children.* Dissertation Abstracts

International: Section B: The Sciences and Engineering. ProQuest Information & Learning, US.

Spence, S.H. (1997). Structure of anxiety symptoms among children: A confirmatory factor-analytic study.

*Journal of Abnormal Psychology, 106*(2), 280-297.

Spielberger, C. D. (1973). *Manual for the State-Trait Anxiety Inventory for Children*. Palo Alto, CA: Consulting

Spirito, Anthony & Williams, Craig & Stark, Lori. (1988). The Hopelessness Scale for Children: Psychometric

properties with normal and emotionally disturbed adolescents. *Journal of Abnormal Child Psychology. 16,* 445-58.

Spirito, A., Stark, L. J., & Williams, C. (1988). Development of a brief coping checklist for use with pediatric

populations. *Journal of Pediatric Psychology, 13*(4), 555–574.

Steele, R. G., Legerski, J.-P., Nelson, T. D., & Phipps, S. (2009). The Anger Expression Scale for Children:

Initial Validation among Healthy Children and Children with Cancer. *Journal of Pediatric Psychology, 34*(1), 51-62.

Stringaris A, Goodman R, Ferdinando S, Razdan V, Muhrer E, Leibenluft E, & Brotman MA. (2012) The

Affective Reactivity Index: a concise irritability scale for clinical and research settings. *Journal of Child Psychology and Psychiatry, 53*(11), 1109–17.

Tennant, Ruth & Hiller, Louise & Fishwick, Ruth & Platt, Stephen & Joseph, Stephen & Weich, Scott &

Parkinson, Jane & Secker, Jenny & Stewart-Brown, Sarah. (2007). The Warwick-Dinburgh mental well-being scale (WEMWBS): Development and UK validation. *Health and Quality of Life Outcomes*, *5*, 63.

Thomaes, Sander & Stegge, Hedy & Bushman, Brad & Olthof, T. & Denissen, Jaap. (2008). Development and

Validation of the Childhood Narcissism Scale. *Journal of Personality Assessment*, 90, 382-91.

Thomas, A., Cairney, S., Gunthorpe, W., Paradies, Y., & Sayers, S. (2010). Strong Souls: Development and

Validation of a Culturally Appropriate Tool for Assessment of Social and Emotional Well-Being in Indigenous Youth. *Australian & New Zealand Journal of Psychiatry, 44*(1), 40–48.

Thompson, L., Reville, H., Price, A., Reynolds, L., Rodgers, L. and Ford, T. (2014) The Quality of Life Scale

for Children (QoL-C), *Journal of Children's Services, 9*(1), 4-17.

Tomyn, A. J., Fuller-Tyszkiewicz, M., & Cummins, R. A. (2013). The Personal Wellbeing Index: Psychometric

equivalence for adults and school children. *Social Indicators Research, 110*(3), 913-924

Viar-Paxton, M. A., Ebesutani, C., Kim, E. H., Ollendick, T., Young, J., & Olatunji, B. O. (2015). Development

and initial validation of the Child Disgust Scale. *Psychological Assessment, 27*(3), 1082–1096.

Victor, S & Klonsky, D. (2016). Validation of a Brief Version of the Difficulties in Emotion Regulation Scale in

Five Samples. *Journal of Psychopathology and Behavioral Assessment, 38,* 582-589.

Walden, Tedra & Harris, Vicki & Catron, Tom. (2003). How I Feel: A Self-Report Measure of Emotional

Arousal and Regulation for Children. *Psychological assessment, 15,* 399-412

Watson, D., & Clark, L. A. (1994). The PANAS-X: Manual for the Positive and Negative Affect Schedule-

Expanded Form. Ames: The University of Iowa

Silverman, W., Fleisig, W., Rabian, B. & Peterson, R. (1991) Childhood Anxiety Sensitivity

Index, *Journal of Clinical Child Psychology, 20*(2), 162-168,

White, B (2014). The Perceived Stress Scale for Children: A Pilot Study in a Sample of 153 Children.

*International Journal of Pediatrics and Child Health, 2,* 45-52.

Yalin Sapmaz, Ş., Özek Erkuran, H., Yalin, N., Önen, Ö., Öztekin, S., Kavurma, C., Köroğlu, E., & Aydemir,

Ö. (2017). Validity and Reliability of the Turkish Version for DSM-5 Level 2 Anger Scale (Child Form for Children Aged 11-17 Years and Parent Form for Children Aged 6-17 Years). *Noro psikiyatri arsivi*, *54*(4), 334–338.

Zeman, J., Shipman, K., & Penza-Clyve, S. (2001). Development and Initial Validation of the Children’s

Sadness Management Scale. *Journal of Nonverbal Behavior, 25*(3), 187-205.
